# Whole-proteome structures shed new light on posttranslational modifications

**DOI:** 10.1371/journal.pbio.3001673

**Published:** 2022-05-27

**Authors:** Robbie P. Joosten, Jon Agirre

**Affiliations:** 1 Biochemistry Department, Netherlands Cancer Institute, the Netherlands and Oncode Institute, Amsterdam, the Netherlands; 2 York Structural Biology Laboratory, Department of Chemistry, University of York, York, United Kingdom

## Abstract

In this new age of accurate structure prediction, calculated protein-only models may show the structural fingerprints of likely post-translational modifications. This Primer explores the consequences of a PLOS Biology study which adds a functional context by combining these with readily available proteomics results.

## Teaser

Accurate but protein-only AlphaFold models may show structural fingerprints of likely posttranslational modifications (PTMs). In this issue of *PLOS Biology*, Bludau and colleagues add a functional context to models by combining them with readily available proteomics results.

The recent artificial intelligence revolution in protein structure prediction, spearheaded by DeepMind’s AlphaFold [1] and swiftly seized upon by RoseTTAFold [2], is allowing scientists to arrive at an accurate structural model of a protein, or at least parts of it, in a matter of hours. This already diminutive lead time can be further compressed to mere seconds if the protein of interest is found in the complete set of proteins expressed by an organism (proteome) in the list of the ever expanding set of organisms covered by the AlphaFold Protein Structure Database (AFDB). The AFDB, released in 2021 and subsequently updated [3], is expected to cover the 100 million set of sequences in the proteomes available at UniRef90 [4]. It offers immediate access to predicted models of human proteins, alongside reliable estimates of their accuracy in the form of 2 metrics: pLDDT (per-residue confidence) and PAE (positional alignment error of each residue with respect to the rest). Structures with a consistently high pLDDT and very low PAE are expected to show an accuracy on par with experimentally determined protein models.

Human proteins are obvious targets for therapeutics; however, their function and structure are, more often than not, modulated or regulated by co- and posttranslational (covalent) modifications, plus ligands and cofactors (noncovalent). Those important moieties, not currently targeted by the AlphaFold algorithm, are conspicuously absent from predicted structures [5]: As an example, many more than half of all human proteins are expected to include either protein glycosylation [6], phosphorylation [7], or both. Thus, the analysis of AlphaFold structures of modified proteins can produce misleading results [5].

Recent studies have suggested that most predicted models are accurate enough to include space for the absent modifications, ligands, and cofactors to be added postprediction [5,8]. Importantly, these endeavours can only be as successful as our ability to pinpoint their occurrence and location on a protein’s structure. In a slightly different case, transplanting likely ligands (e.g., a heme group onto hemoglobin or a polysaccharide onto a glycoside hydrolase) onto AlphaFold models by homology with experimental structure models becomes increasingly error-prone when the homology becomes more distant. In the case that homology is absent altogether, transferable knowledge from experimental structure models is absent as well, and this process becomes a speculative docking experiment.

In the absence of experimental structural models, the extensive proteomics datasets available today can provide information on co- and posttranslational modifications (PTMs) on the respective target proteins [9]. Furthermore, the covalent transference of modifications onto protein often follows a consensus sequence—e.g., N-glycosylation on Asn-X-Ser/Thr where X is any amino acid other than proline; these consensus sequences are variably well studied across modifications. Crucially, mapping proteomics and bioinformatics information onto AlphaFold 3D models may allow us to not just complete models, but to learn more about the structural fingerprints left by modifications: the structure of their protein scaffold and their environment. In this issue, Bludau and colleagues [10] discuss the first results from the implementation of such an approach, targeting different modification types including phosphorylation, ubiquitination, and more.

**Fig 1:**
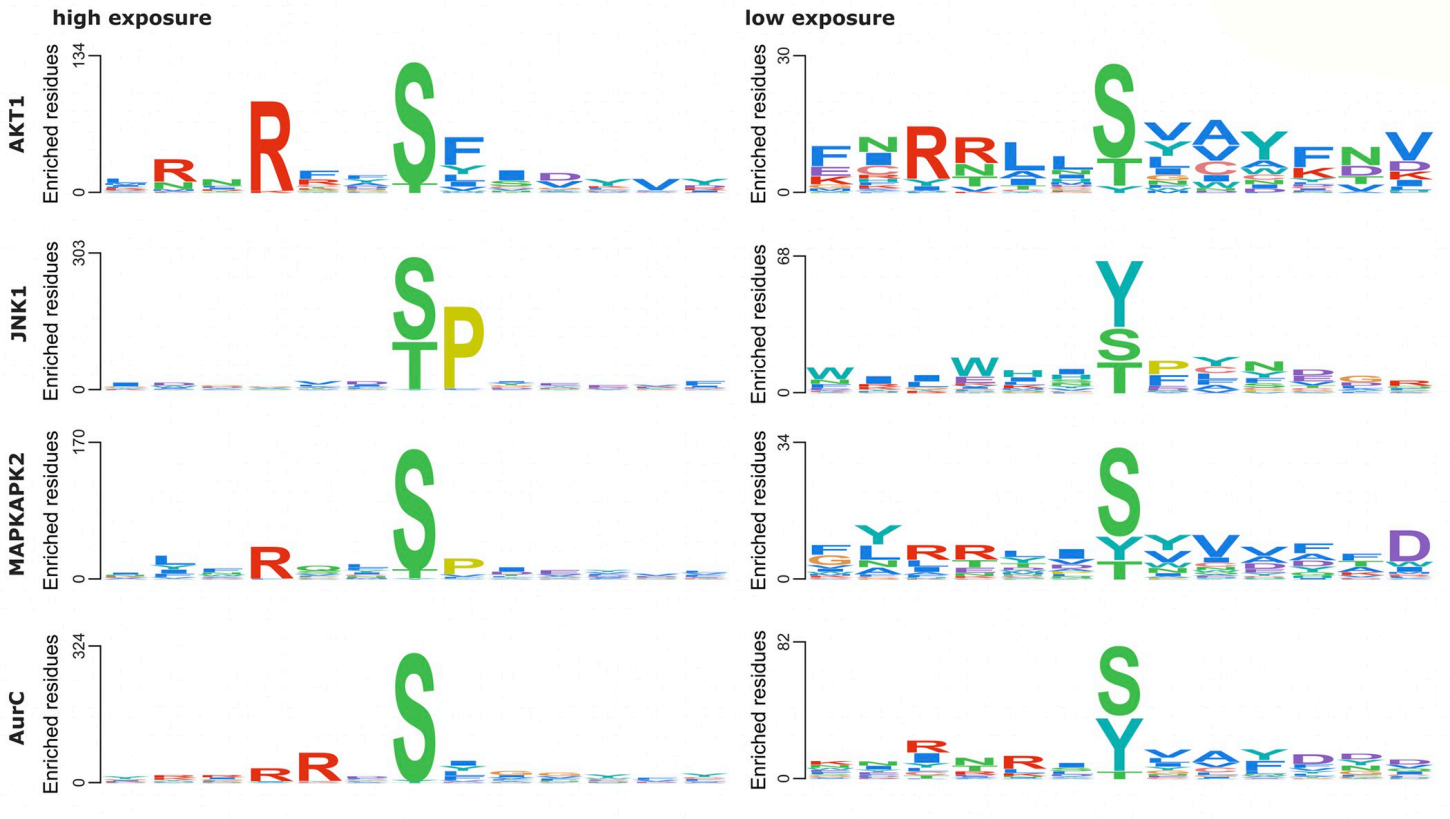
Different sequence profiles in exposed and solvent-excluded phosphosites (STY) as recognised by different kinases. The availability of accurate 3D models now allows for this direct mapping of sequence profiles onto structures and allows estimating their solvent accessibility. Extracted from Fig 3d of Bludau and colleagues [10].

Not all PTMs are made equal: They may play different roles depending on whether they are buried or exposed to solvent (Fig 1), added to a correctly folded region, a misfolded region, or to an intrinsically disordered one. On that last point, the synergy with AlphaFold brings another important contribution to the table: Because AlphaFold has been trained on data from the structured parts of ordered proteins—a precondition for atomic positions to be well resolved in both X-ray crystallography and electron cryo-microscopy, the 2 main techniques contributing structures to the Protein Data Bank (PDB)—there is a good correlation between intrinsic disorder and low prediction confidence as measured by AlphaFold;s pLDDT [1]. Bludau and colleagues [10] use this knowledge to select PTMs that are enriched for having regulatory functions. These regulatory modification sites show a preference for short intrinsically disordered regions such as the activation loops in protein kinases. In addition, the authors use AlphaFold models to show that different regulatory modification sites have a strong tendency to flock together in 3D and not just in sequence space, hinting at coregulation or even cross talk between different types of PTMs [10].

The work, as one of the first systematic analyses of the functional importance of PTMs, lays an important foundation for new experimental studies targeting PTMs in specific proteins. The authors provide software tools to shortlist the modification sites of regulatory importance, thereby allowing more focused experimental studies. Importantly, the software—for which source code is available from the “structuremap” and “alphamap” repositories at https://github.com/MannLabs—will also enable richer annotation of PTMs on AlphaFold entries. To this end, we think the results from Bludau and colleagues [10] would make a worthy contribution to the recently introduced 3D-Beacons database, which aims to become a reference point for structural knowledge (https://www.ebi.ac.uk/pdbe/pdbe-kb/3dbeacons).

## Abbreviations

AFDB: AlphaFold Protein Structure Database
PAE: positional alignment error
PDB: Protein Data Bank
PTM: posttranslational modification
